# Children’s Self-Perceived and Actual Motor Competence in Relation to Their Peers

**DOI:** 10.3390/children5060072

**Published:** 2018-06-08

**Authors:** Ryan Washburn, Angela Kolen

**Affiliations:** Department of Human Kinetics, St. Francis Xavier University, 1140 Convocation Blvd, Antigonish, NS B2G 2W5, Canada; ryanwashburn12@gmail.com

**Keywords:** physical literacy, physical competence, physical activity, physical activity promotion

## Abstract

Motor skill competence enables children to move with efficiency and confidence in a variety of physically challenging situations. A child who lacks motor skill competence may be less inclined to take part in physical activities in which his or her peers excel. In this regard, the development of motor competence and children’s perception of their motor abilities may play an important role in ensuring sufficiently physically active adults. To better understand the role of motor competence in children’s participation in physical activity, this study examined children’s perception of their motor competence in comparison to others with their actual motor competence. Data were collected from 1031 children in grades 3, 4, 5, and 6 and between the ages of 8 to 12 years from elementary and junior schools. Using the Canadian Assessment of Physical Literacy (CAPL) protocols, physical competence and perceived physical competence were obtained from the Canadian Agility and Movement Skill Assessment and the CAPL questionnaire, respectively. Results from this study support previous research as children’s ability to accurately perceive their motor competence increased with age/grade. Still, over half of the participants in this study were not able to accurately perceive their motor competence. In addition, as grade increased from 3 to 6, children over-estimated their abilities less and underestimated their abilities more. This lack of ability to accurately estimate their abilities may be impacting children’s level of physical activity and should be addressed when promoting physical activity.

## 1. Introduction

Physical inactivity has become an increasing source of disease and premature death among Canadians. Children today continue to be insufficiently physically active with only eight percent of Canadian children, ages 8 to 12 years, meeting the recommended amount of daily physical activity [1]. In addition, only 24% of children of ages 5 to 17 years meet the recommended sedentary time restrictions per day [1]. With these findings, it is important to identify why childhood physical activity levels are alarmingly low and why children participate in a high level of sedentary behaviours.

A potential explanation for this low level of childhood physical activity may relate to motor skill competence [2], a component of physical literacy. Physical literacy is defined as the motivation, confidence, physical competence, knowledge and understanding to maintain physical activity throughout the life course [3]. A child with a lower level of physical or motor competence may be less inclined to take part in physical activities in which his or her peers excel [4]. Caput-Jogunica et al. [5] examined the influence of extra-curricular sports programs on the motor abilities and physical literacy of preschool aged children (four–six years). Using several physical tasks to examine motor abilities (explosive strength, dynamic strength, coordination, flexibility and balance), it was found the development of sufficient motor proficiencies in early childhood positively correlated with club sport participation and total time spent in sport later in life [6]. Therefore, it is imperative that children develop their motor skills at a young age so they grow to become physically skilled, capable and physically active individuals. Further, the perception of motor abilities may also play an important role in children’s level of physical activity participation and in their physical literacy [2]. If children perceive themselves as having high levels of motor competence, they are more likely to participate in more physical activities. Alternatively, if children perceive themselves as having low levels of motor competence, it is believed they will not choose to participate in physical activity [2].

Motor competence, a component of physical literacy, has been shown to greatly impact an individual’s level of physical activity. Khodaverdi et al. [7] examined actual and perceived motor competence and physical activity in children using the Test of Gross Motor Development-2 to assess actual motor competence and the Self-Description Questionnaire-1 to evaluate perceived motor competence. It was found that barriers to physical activity included low levels of actual and perceived motor competence, low health-related physical fitness levels, and obesity [7]. In contrast, Fransen [6] measured motor competence using the Körperkoordinations Test für Kinder and stated, “children with a higher motor competence choose to participate in a more varied range of physical activities, giving them the opportunity for a variety of movement experiences which might in turn result in greater amounts and intensity of physical activity” (pg. 12). It is thought that children who possess a higher level of motor competency are better able to cope with the demand of participating in sporting activities and may therefore be more physically active through sport, gaining additional physical and mental health benefits [6]. In addition, it is believed that sufficient motor competence promotes a synergistic relationship with physical activity, which may increase positive and healthy behaviours [7].

Children are believed to be able to make judgements about their physical competence after the age of 8 [8]. As such, prior to this age, children are not likely to recognize their actual physical competence and do not begin to compare their abilities with peers and become more aware of their motor skill performance until they are older [9]. This shift in perception is usually made within a public setting (physical education, organized sport, etc.) and leaves children susceptible to humiliation and embarrassment when they believe their motor skill performance is not as good as others. Kirk found that children quickly learned their place among peers in relation to their abilities in the classroom, friendship groups and in physical activities, including sport [10]. In this regard, it is essential that children develop positive feelings toward physical activity early in life—including their competence in relation to their peers to set a foundation for a physically active lifestyle in the future [11]. Further, in most physical activity or sport settings, little if anything is done to encourage participation for those children who perceive their motor competence as low [12]. As such, many children may no longer wish to participate in physical activity due to fear of embarrassment when putting their motor skills on display. It is thought that increasing positive self-perceptions of motor skill performance in children may be important for facilitating participation in sport and physical activity [2,13].

Thus, to better understand the role of motor competence in children’s participation in physical activity, this study examined children’s perception of their motor competence in comparison to others with their actual motor competence. It was hypothesized that at a young age (≤9 years or in grades 3 or 4), children would not accurately perceive their motor competence and at an older age (>9 years or in grades 5 or 6), children would be more accurate in perceiving their motor competence.

## 2. Materials and Methods

Data used in this study came from one of the data collection sites of a larger research project, the Canadian Assessment of Physical Literacy (CAPL). Following pilot-testing of the data collection protocol, the national CAPL research project was led by the Healthy Active Living and Obesity (HALO) Research Group at the Children’s Hospital of Eastern Ontario (CHEO) with several data collection sites across Canada. More details can be found from the CAPL website (www.capl-eclp.ca). Research ethics approval was obtained from the CHEO (protocol number: 13/202X) as well as St. Francis Xavier University (protocol number: 21990) and the school board from where the students were invited to participate.

### 2.1. Participants

Children in grades 3, 4, 5, and 6, and between the ages of 8 and 12 years were recruited from local elementary and junior schools. Following a letter of invitation given to potential participants in the school setting, parents/guardians provided written consent and the children themselves provided verbal assent at the time of data collection. If the child did not provide assent, this outweighed the decision of the child’s parent/guardian. As part of the informed consent, parents/guardians completed a ‘health check’ questionnaire to identify any medical conditions that might hinder their child’s participation in the physical tasks of the study (i.e., the Canadian Agility and Movement Skill Assessment (CAMSA) and other physical tasks not included in this study).

### 2.2. Experimental Design

Data collection for this study followed a cross-sectional research design. Data were most often collected from participants during one school visit. For this study, data were collected using the Canadian Agility and Movement Skill Assessment (CAMSA) and a self-report questionnaire. This study required the assistance of several data collectors due to the large number of children participating at one time. All data collectors attended training sessions which included opportunities for practical experience with data collection techniques to enhance consistency among assessments. Where possible, the same person collected a particular component of the CAPL data collection.

### 2.3. Protocol

To measure the actual level of motor competence, the CAMSA was completed [14]. To measure perceived levels of motor competence, the CAPL questionnaire was completed [15].

### 2.4. Actual Motor Competence

To measure motor competence, each child completed CAMSA. Prior to the child beginning the CAMSA, the test administrator demonstrated how to complete the CAMSA twice to familiarize the child with it. During the first demonstration, the test administrator moved through the assessment slowly and completed every physical task perfectly while verbally describing the skill and emphasizing cue words. According to the CAPL protocol, the second demonstration involved the administrator completing the CAMSA as quickly as possible, while maintaining correct skill performance and accuracy to emphasize the importance of completing the course as fast as possible. Following this verbal and visual demonstration, each child completed the CAMSA. Following their practice, two trials were timed and scored by the test administrators. For a complete description of the CAMSA, please see the Canadian Assessment of Physical Literacy manual which can be accessed at www.capl-ecsfp.ca/capl-manual/.

Two data collectors obtained the CAMSA data. In addition to being the timer, data collector number one provided standard verbal cues for each skill for each child according to CAPL protocols to ensure the score and time reflected motor skill ability rather than memory. Data collector number two scored the child’s motor skill performance. Each child’s CAMSA performance was scored out of 14, with one point awarded for each skill performance criterion. These criteria were scored as 1 = done correctly or 0 = done incorrectly, with no part marks given. Time was converted into a score out of 14 and coupled with the skill score to provide a CAMSA score out of 28. 

### 2.5. Perceived Motor Competence

Part of the CAPL assessment involved the administration of a questionnaire to measure knowledge, understanding, motivation, and confidence towards physical activity [15]. Within this questionnaire designed specifically for the CAPL project, children were asked to compare themselves to others their age in regard to their motor skill performance. Specifically, the children were asked, “Compared to other kids your age, how good are you at sports or skills?” Response choices were on a Likert scale from 1–10, with 1 being “others are a lot better”, 10 being “I’m a lot better” and 5 or 6 indicating a “similar level of performance”.

### 2.6. Statistical Analysis

To determine the relationship between actual motor competence and perceived motor competence, simple descriptive analyses were conducted. Average questionnaire scores were determined for participants in each grade (i.e., 3, 4, 5, and 6). Then, each participant’s self-reported or perceived motor competence score (i.e., 1–10 in response to “Compared to other kids your age, how good are you at sports or skills?”) was compared to his/her actual motor competence performance (i.e., CAMSA score) and the average CAMSA score calculated for all participants in their grade. Each participant was then given a rating of “underestimated”, “accurately estimated”, or “overestimated” depending upon the discrepancy, or lack thereof, of these measures. 

When obtaining individual questionnaire scores, ratings of below average, average and above average were given to each participant. If participants responded with an answer between 1–3, it was interpreted that these children believed they possessed lower levels of skill compared to their peers and were given a rating of “below average”. A response of 4–7 was given the rating “average” and a response of 8–10 corresponded to “above average”. The same rating scale was used for participants’ levels of actual motor competence. If a participant’s CAMSA score fell within one standard deviation above or below their respective grade average, they were assigned the rating of “average”, if their score was below one standard deviation they were rated as “below average” and subsequently “above average” if their score was above one standard deviation.

## 3. Results

Although there were 1162 participants from this data collection site for the CAPL project, for inclusion in this data analysis, participants required objective and subjective measures of their motor competence. In other words, they needed to have completed two trials of the CAMSA and answered the following from the questionnaire: “Compared to other kids your age, how good are you at sports or skills?” With this criterion, data were available for 1031 or 88.7% of participants with their grade distribution shown in Table 1.

### Data Management 

For analysis in this study, individual CAMSA scores were determined from participants’ second trial composite scores (time to complete the CAMSA plus skills score). As per the CAPL manual, participants’ individual CAMSA scores were not averaged as there is potential for a learning effect when completed fewer than three times. The participants’ CAMSA composite scores were then averaged with respect to grade to determine the average score for participants according to grade. 

Using the categorizations previously described in the statistical analysis section, the number of participants in each category according to grade for actual ability is presented in Table 2.

Using the categorizations previously described in the statistical analysis section, the categorization of the participants according to grade for perceived motor skills ability is presented in Table 3.

Based on the categorization of perceived and actual motor skill abilities, participants were then assigned a word combination which indicated if they over perceived, under perceived or accurately perceived their motor competence as shown in Table 4.

Following the assignment of actual perceptions, the percent of individuals in each grade who were categorized as ‘accurate’, ‘underestimate’ or ‘overestimate’ was determined. The number and percentage of students in each category according to grade are presented in Table 5.

**Figure 1:**
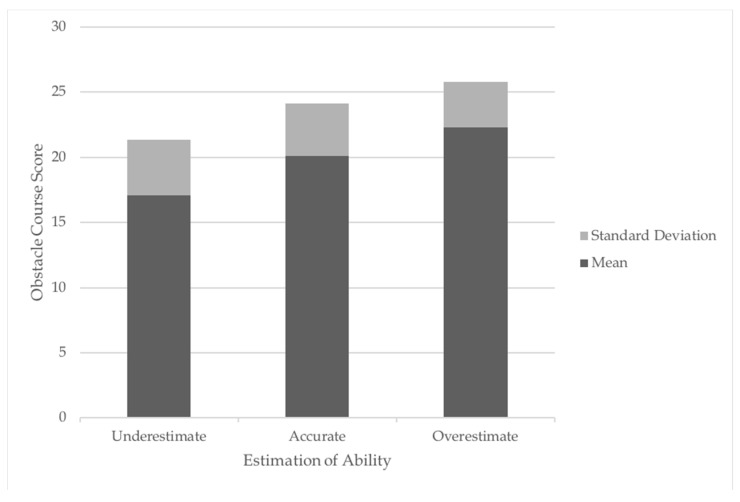
Average (SD) CAMSA scores of participants who under, accurate, or overestimated their motor competence.

A one-way analysis of variance (ANOVA) was then performed to determine if there was a significant difference between accurate, under and over perception groups in CAMSA performance. Since a significant difference (*p* = 0.000) was found, a Tukey post-hoc test was performed to determine where the difference existed. This post-hoc analysis indicated significant differences between participants that underestimated and accurately estimated (*p* = 0.15) their abilities as well as between those who overestimated and accurately estimated their abilities (*p* = 0.000). More specifically, participants that accurately estimated their abilities had, on average significantly lower CAMSA scores (20.1 ± 4.03) than children that overestimated (22.3 ± 3.47) their abilities and significantly higher scores than children who underestimated their abilities (17.1 ± 4.27). (See Figure 1). 

## 4. Discussion

The purpose of this study was to determine how well children perceive their motor competence. More specifically, this study compared actual and perceived motor competence in children in grades 3 to 6. It was hypothesized that at a young age (≤9 years or in grades 3 or 4), children would not accurately perceive their motor competence and at an older age (>9 years or in grades 5 or 6), children would be more accurate in perceiving their motor competence. This hypothesis was supported as results indicated younger children tended to overestimate their abilities while older children were better able to accurately estimate their abilities. More specifically, 72.4% of students in grade 3 under or overestimated their motor abilities while 49.8% of students in grade 6 accurately estimated their motor competence. These results indicated that older children are better at recognizing their motor competence in comparison to others, however, it is important to note, more than half (56.7%) of the participants in this study overall did not accurately perceive their motor competence.

As previously mentioned, throughout Canada, children are not sufficiently physically active, with only eight percent meeting the recommended daily physical activity guidelines [16]. Physical literacy may play a role in children’s decisions to be physical active with higher levels of physical literacy related to higher levels of physical activity. This may be in part due to motor competence—a component of physical literacy which is believed to influence physical activity participation [2]. The inability to perceive motor competence is potentially a barrier to physical activity participation [7]. Although levels of physical activity among children in Nova Scotia are low, the children in this study possess sufficient motor competence [3]. The discrepancy in physical activity levels and motor competence may be attributed to the fact that over half (56.7%) of the participants inaccurately perceived their motor competence. Although accuracy of perceptions did increase as children became older, the majority of participants were not able to accurately perceive their motor competence. Children in grade 6 showed the most accuracy (49.8%), while children in grade 3 were the least accurate (27.5%) in recognizing their motor competence. In addition, from grade 3 to 6, children increasingly underestimated their level of motor competence. 

As children age, their self-perceptions begin to shift. It is believed that after age nine, children begin to compare themselves to their peers and quickly learn their place among peers in relation to abilities in the classroom, friend groups and physical activities such as sports [9]. This shift in perception usually takes place in public settings such as physical education classes, organized sport and other group physical activities. Some children may feel they possess inadequate motor skills when they think their skills are not as good as their peers. The current study showed a large shift in perception as children in grade 3 (8.5 ± 0.47 years) tended to overestimate their motor competence with 67.7% reporting they were better at performing skills than their peers when, in reality, they were performing similarly or not as well as their peers. This overestimation may be attributed to the belief that these younger children are not able to make discrete judgments about their physical competence [8]. In contrast to this finding, almost one-third of children in grade 6 (11.7 ± 0.42 years) underestimated their motor competence with 30.4% of children self-reporting they were not as good at sport skills when compared to their peers, when they actually were average or above average in their skill performance. This shift in perception causes concern as children are less likely to participate in physical activities if they believe they are not as good as their peers [2]. As such, it is imperative to further investigate this phenomenon to better understand it as well as to find ways to decrease this negative perception shift so that children are motivated to participate in a variety of physical activities without the stress of embarrassment and instead with feelings of confidence and competence. In this regard, it is important children are made aware of individual levels of ability. This awareness may be heightened by increasing discussions regarding individual motor competence without comparing peers. In doing this, inaccurate estimations of individual motor competence may be decreased and the space to be physically active may feel safe and more conducive to the trials and errors of developing motor skills. 

Throughout this study several limitations arose. Although all data collectors participated in several training sessions regarding data collection, variability may have existed among data collectors for the CAMSA. Inter or intra-rater related reliability among and between the data collectors for the CAMSA was not determined. Similarly, although all data collectors were instructed to provide consistent motivation to the children completing the CAMSA, it is difficult to ascertain the impact of different data collectors. In addition, participants’ comprehension, or lack thereof, of the question used, may have led to inaccurate answers. Some participants, especially those in grade 3, may not have fully understood the question or may have circled an answer without fully comprehending it. In addition, in some instances, participants answered the question while sitting next to a friend and may have been influenced by their peers when answering the question. Further, a more reliable estimate of perceived motor competence should be obtained from more than one question. A strength of this study was the large data set used with 1031 of 1162 possible participants (i.e., 88.7%) included in the analysis. Another strength was the use of the CAMSA, a reliable and valid indicator of motor competence related to physical literacy.

## 5. Conclusions

The results of this study indicate that the majority of children are unable to accurately perceive their motor abilities even though their ability to accurately perceive their motor skill abilities increases with age. Participation in physical activity may be heightened if accuracy of motor ability perceptions is increased and negative perceptions are decreased given the potential connection between self-confidence in motor abilities and physical activity. As a society, we must ensure children possess the competence, confidence and knowledge about movement skills to obtain sufficient physical activity throughout life. 

## Figures and Tables

**Table 1:** Number of children that provided complete Canadian Agility and Movement Skill Assessment (CAMSA) and questionnaire data. CAPL: Canadian Assessment of Physical Literacy.

Grade	Children with CAPL Data	Children with CAMSA and Questionnaire Data
3	135	127
4	520	443
5	242	214
6	265	247
Total	1162	1031

**Table 2:** Categorization of participants according to CAMSA ability.

Grade	Above *	Average **	Below ***
**3**	6	78	43
**4**	43	316	84
**5**	39	155	20
**6**	96	134	17

* above = CAMSA score equal to or exceeds one standard deviation (SD) of grade mean; ** average = CAMSA score within one SD of grade mean; *** below = CAMSA score equal to or less than one SD of grade mean.

**Table 3:** Categorization of participants according to perceived motor skills ability and grade.

Grade	Above *	Average **	Below ***
**3**	73	43	11
**4**	209	190	44
**5**	65	120	29
**6**	82	134	31

* above = response of 7–10 on the Likert scale; ** average = response of 4–7 on the Likert scale; *** below = response of 1–3 on the Likert scale.

**Table 4:** Combining children’s perception of ability and actual ability categorizations.

Perception of Ability	Actual Ability	Word Combination	Actual Perception
Average	Average	Average + Average	Accurate
Below	Below	Below + Below	Accurate
Above	Above	Above + Above	Accurate
Below	Average	Below + Average	Underestimate
Average	Above	Average + Above	Underestimate
Below	Above	Below + Above	Underestimate
Average	Below	Average + Below	Overestimate
Above	Average	Above + Average	Overestimate
Above	Below	Above + Below	Overestimate

**Table 5:** Number (and percentage) of children assigned to each category of perception according to grade.

Grade	Accurate Perception	Over Perceive	Under Perceive	Total
**3**	35 (27.5%)	86 (67.7%)	6 (4.7%)	127
**4**	185 (41.7%)	220 (49.6%)	38 (8.5%)	443
**5**	103 (48.1%)	61 (28.5%)	50 (23.3%)	214
**6**	123 (49.8%)	49 (19.8%)	75 (30.4%)	247
